# A transgenic system for generation of transposon *Ac/Ds*-induced chromosome rearrangements in rice

**DOI:** 10.1007/s00122-012-1925-4

**Published:** 2012-07-14

**Authors:** Chuanhe Yu, Fangpu Han, Jianbo Zhang, James Birchler, Thomas Peterson

**Affiliations:** 1 Department of Genetics, Development and Cell Biology, Department of Agronomy, Iowa State University, Ames, IA 50011 USA; 2 Division of Biological Sciences, University of Missouri, Columbia, MO 65211 USA; 3 State Key Lab of Plant Cell and Chromosome Engineering, Institute of Genetics and Developmental Biology, Chinese Academy of Sciences, 100101 Beijing, China

## Abstract

The maize *Activator* (*Ac*)/*Dissociation* (*Ds*) transposable element system has been used in a variety of plants for insertional mutagenesis. *Ac/Ds* elements can also generate genome rearrangements via alternative transposition reactions which involve the termini of closely linked transposons. Here, we introduced a transgene containing reverse-oriented *Ac/Ds* termini together with an *Ac* transposase gene into rice (*Oryza sativa* ssp. *japonica* cv. *Nipponbare*). Among the transgenic progeny, we identified and characterized 25 independent genome rearrangements at three different chromosomal loci. The rearrangements include chromosomal deletions and inversions and one translocation. Most of the deletions occurred within the T-DNA region, but two cases showed the loss of 72 kilobase pairs (kb) and 79 kb of rice genomic DNA flanking the transgene. In addition to deletions, we obtained chromosomal inversions ranging in size from less than 10 kb (within the transgene DNA) to over 1 million base pairs (Mb). For 11 inversions, we cloned and sequenced both inversion breakpoints; in all 11 cases, the inversion junctions contained the typical 8 base pairs (bp) *Ac/Ds* target site duplications, confirming their origin as transposition products. Together, our results indicate that alternative *Ac/Ds* transposition can be an efficient tool for functional genomics and chromosomal manipulation in rice.

**Electronic supplementary material:**

The online version of this article (doi:10.1007/s00122-012-1925-4) contains supplementary material, which is available to authorized users.

## Introduction

With the completion of several plant genome sequencing projects (Arabidopsis Genome Initiative 2000; International Rice Genome Sequencing Project 2005), a major goal remaining in plant genome research is to determine the functions of individual genes and gene families. Two mutagenesis methods have been widely used to generate the loss-of-function alleles. One method employs chemical mutagens, such as ethyl methanesulfonate (EMS) (Hirochika et al. 2004). One disadvantage of chemical mutagenesis is that multiple independent mutations are commonly generated, and several generations of backcrossing may be needed to separate the desired mutation from others in the genome. In addition, the mapping and molecular isolation of genes containing EMS-induced mutations are often laborious and time consuming. A second method employs T-DNA or transposable elements for gene tagging (Miyao et al. 2003; Sallaud et al. 2003). T-DNA and transposon insertion sites can be easily mapped and isolated, but the generation of mutant collections containing sufficient numbers of insertions in large complex genomes is often challenging.

While chemical and insertional mutagenesis methods are useful for single-gene targets, they are not generally applicable for generating more extensive changes. Physical agents such as ionizing radiation can induce rearrangements including deletions, inversions, and translocations (Cecchini et al. 1998). However, this method has not been widely used in recent years because the random breakpoint locations can render the products somewhat difficult to analyze. Another chromosome rearrangement tool uses *Ac/Ds* transposable elements in combination with the Cre/lox site-specific recombination system (Medberry et al. 1995; Osborne et al. 1995; Stuurman et al. 1996). The approach involves a number of steps: (1) plants are transformed with a construct containing a mobile *Ds* element harboring a lox locus; (2) the transformed plants are crossed with an *Ac* transposase source line to induce *Ds* transposition; (3) plants containing transposed *Ds* elements are crossed with a line expressing Cre recombinase to induce deletion or inversion of the chromosome segment between the transposed *Ds* element and the original transgene insertion. This approach has the disadvantage that several plant generations are required before the desired rearrangements can be detected. Additionally, one must map a potentially large number of individual *Ds* insertion sites to identify lines containing *Ds* insertions at the desired locus which are to be crossed with the Cre recombinase.

We have developed an alternative approach for plant genome modification based on the process of alternative transposition, i.e., transposition events which involve one end from each of two different transposons (Zhang et al. 2006). Previously we have shown that alternative transposition of a closely apposed pair of directly oriented *Ac/Ds* termini can lead to the formation of chromosome deletions and inverted duplications, or chromosomal breakage (Yu et al. 2010b; Zhang and Peterson 1999). Using this reaction, we isolated a series of nested deletions flanking the *p1* gene on maize chromosome 1, ranging up to 4.6 cM in size (Zhang and Peterson 2005). Another type of alternative transposition reaction involving reverse-oriented *Ac/Ds* termini can generate deletions, inversions, and translocations (Huang and Dooner 2008; Zhang and Peterson 2004; Zhang et al. 2009). These results arising from natural configurations of *Ac/Ds* elements in maize prompted us to test whether alternative transposition could be reproduced in transgenic systems for functional genomic purposes. The potential advantages of *Ac/Ds*-induced alternative transposition as a mutagenic tool include: (1) deletions can remove multiple copies of clustered genes, thereby simplifying the identification of individual gene functions; (2) it is relatively easy to clone the rearrangement breakpoint sequences by PCR-based methods; (3) a single locus capable of undergoing alternative transposition reactions can generate a broad spectrum of possible products; (4) *Ac/Ds* exhibits a preference for local transposition, thereby enriching the rearrangements in the targeted genome regions; (5) rearrangements such as inversions and translocations may be useful for manipulating chromosome structure and for the detection and analysis of chromosome-level influences on gene expression, e.g., position effect. In a previous work, an *Ac/Ds* alternative transposition-based system generated a variety of rearrangements in Arabidopsis, thus validating the principle. However, most of the rearrangement events obtained appeared to be somatic, apparently due to the inefficiency of the selection markers used (Krishnaswamy et al. 2008).

Here, we describe the development of an alternative transposition-based approach for generating genome rearrangements in rice. Our system utilizes a transgene construct containing a pair of *Ac/Ds* termini in reverse orientation, together with suitable marker genes for the detection of rearrangements. A variety of chromosomal rearrangements were isolated, and the junctions were cloned and sequenced; all of the events obtained were found to have the characteristic features of *Ac/Ds* transposition-induced events. We conclude that alternative transposition can be a useful tool for genome manipulation in rice.

## Materials and methods

### Construction of *pRAc* vector

The 6.8 kb *Ds* element from activation-tagging *Ac*–*Ds* vector *pSQ5* (Qu et al. 2008) was PCR amplified; the final product was designed to include a 50 bp deletion at the *Ds* 3′ end. The terminal deletion derivative *Ds* was then used to replace the original *Ds* element of *pSQ5* in reversed orientation. Because the *pSQ5* construct contains a 5′ terminally truncated *Ac* element, the reverse-oriented *Ac* 3′ end and *Ds* 5′ end constitute the only intact *Ac/Ds* termini in the construct. The final *pRAc* construct was partially sequenced to confirm the junctions of cloned fragments and *Ac/Ds* termini sequences.

### Plant transformation and screening of transgenic plants

The *pRAc* plasmid was transformed into Agrobacterium strain *EHA105*. Rice (*Oryza sativa* ssp. *japonica* cv. *Nipponbare*) callus induction and transformation were performed by the Iowa State University Plant Transformation Facility (http://www.agron.iastate.edu/ptf/). For screening, mature dried seeds of transgenic plants were germinated at 25 °C for 3–5 days on 1/2 MS medium with or without 50 mg/L hygromycin. The emerging seedlings were screened for GFP fluorescence using a dissection microscope (SHZ10, Olympus Co., Japan).

### Genomic DNA extractions, Southern blot hybridization

Young leaves of individual plants were ground in liquid nitrogen, and genomic DNA was extracted with cetyltrimethylammonium bromide (CTAB) reagent Saghai-Maroof et al. (1984). Agarose gel electrophoresis and Southern blot hybridizations were performed according to (Sambrook et al. 1989), except that hybridization buffers contained 250 mM NaHPO_4_, pH 7.2, and 7 % SDS and wash buffers contained 20 mM NaHPO_4_, pH 7.2, and 1 % SDS. DNA probes (7P3P, 69P, and UBIP; Supplementary dataset 1) used for Southern blot were amplified directly by PCR. The primary PCR product with M9-3P probe primers (Supplementary dataset 1) was cut by *Bam*H1 and *Hin*p1I. The resulting 0.5 kb fragment was used as M9-3P probe. DNA fragments used for probes were purified using a Qiagen PCR or gel purification kit (Hilden, Germany). Oligonucleotide probes for Southern hybridizations were labeled by [α-^32^P]dCTP using Amersham Pharmacia Rediprime™ II DNA Labeling System (Piscataway, NJ, USA).

### Polymerase chain reaction analysis

PCR was performed using HotMaster Taq polymerase (Eppendorf, Hamburg, Germany); each reaction used approximately 20 ng of ligated DNA or genomic DNA as template. PCR was performed with an initial denaturation at 94 °C for 3 min, followed by 35 cycles (each cycle at 94 °C for 20 s, 58 °C for 30 s, and 65 °C for 1 min per 1 kb length of expected PCR product) with final at 65 °C for 10 min.

### Isolation of sequences flanking T-DNA insertions or rearrangement breakpoints

Sequences flanking T-DNA insertions and rearrangement breakpoints were isolated by inverse-PCR (Ochman et al. 1988) using the oligonucleotide primers shown in Supplementary dataset 1. About 1 μg of genomic DNA was digested with *Hpy*CH4IV or *Msp*I (NEB, Beverly, MA, USA). Samples were digested overnight, ethanol precipitated, dissolved in water, and self-ligated in a 400 μL volume containing 10 Weiss Unit ligase (NEB, Beverly, MA, USA) at 4 °C for 12 h.

### Fluorescence in situ hybridization (FISH) and immunostaining

Probes used for FISH analysis were amplified directly from genomic DNA by PCR (see Supplementary dataset 1 for primer sequences). Chromosome preparation, FISH, image capturing, and image processing were performed as described (Han et al. 2006; Kato et al. 2004).

## Results

### Transgene construct and transgenic rice starter lines

**Fig. 1:**
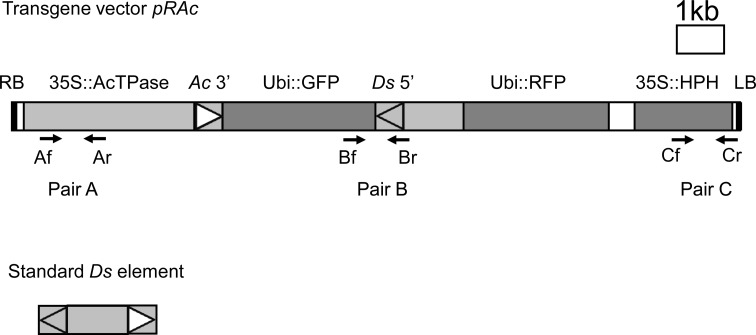
T-DNA region of the transgene vector *pRAc.* *Upper*, structure of the transgene vector *pRAc*. RB and LB, *Right* and *left* *borders* of the T-DNA; *35S* CaMV 35S promoter, *Ubi* maize *ubiquitin* 1 promoter, *GFP* green fluorescence protein gene used as a negative screening marker, *RFP* red fluorescence protein gene used as a positive screening marker to indicate the presence of transgene, *HPH* hygromycin phosphotransferase gene used as a plant transformation selection marker and a positive screening marker for the presence of the transgene. All genes shown are in the same transcriptional orientation (*left* to *right*). The T-DNA is within the *pCAMBIA*-*1300* backbone. The intact *Ac*/*Ds* 3′ and 5′ termini are shown; other deleted non-functional *Ds* termini are not shown. The positions of PCR primer pairs (Af/Ar, Bf/Br; Cf/Cr) are marked on the construct. *Lower*, structure of a standard *Ds* element. The *Ds* 5′ and 3′ termini are indicated by the *shaded* and *open arrowheads*, respectively

A transgenic construct (*pRAc*) designed to undergo alternative transposition is shown in Fig. 1. The construct contains a hygromycin-resistance gene (*HPH*) driven by the CaMV 35S promoter for positive selection. In addition, a synthetic green fluorescent protein (*GFP*) gene driven by the maize ubiquitin 1 (UBI) promoter was used as a negative screening marker (Jeon et al. 2000; Jeong et al. 2002; Kolesnik et al. 2004; Kumar et al. 2005). To eliminate the need for crossing with an *Ac* transposase source, the construct contains a 5′-truncated *Ac* element driven by the CaMV 35S promoter (Qu et al. 2008). The 3′ terminus of the truncated *Ac* element is in reverse orientation with respect to the 5′ end of a 3′-truncated *Ds* element. The GFP marker is located between the *Ac* 3′ end and the *Ds* 5′ end. In this configuration, alternative transposition events involving the reversed *Ac/Ds* termini will result in the loss of GFP. In addition, the construct contains a *RFP* gene located downstream of the 5′ *Ds* terminus that can be used as a positive screening marker (Fig. 1).

**Fig. 2:**
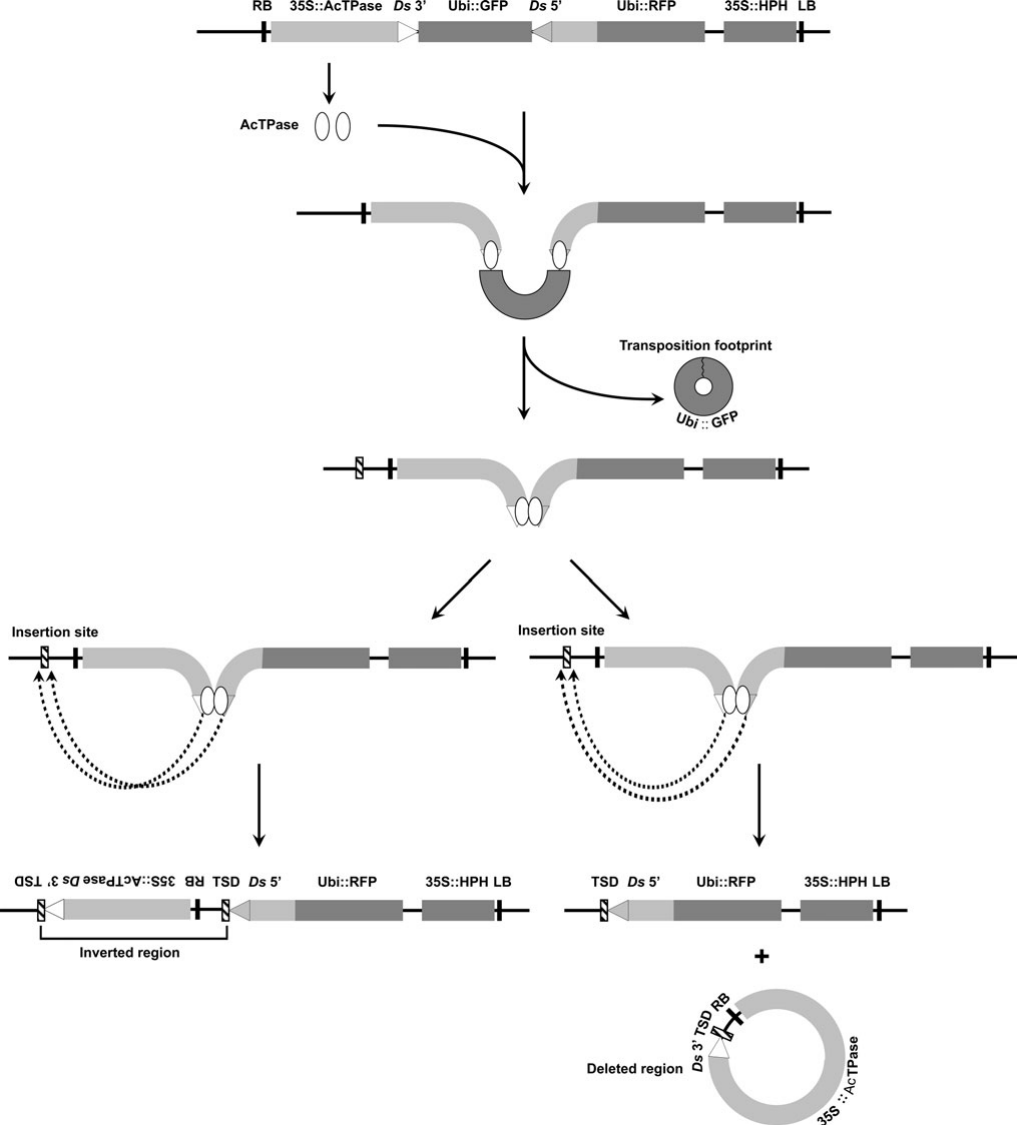
Models for the formation of inversions or deletions by alternative transposition of reversed *Ac*/*Ds* termini. *Ac* transposase expressed from the transgene construct recognizes and excises the reverse-oriented *Ac*/*Ds* termini. The inter-transposon segment (ITS) containing UBI::GFP forms a circle which is subsequently lost. The excised *Ac*/*Ds* ends in a complex with *Ac* transposase will integrate into the genome. Integrations on the same chromosome result in either inversion (*left*) or deletion (*right*), depending on the orientation in which the *Ac*/*Ds* termini integrate. Note that inversion chromosomes contain both copies of the TSD generated upon integration, whereas in deletion events one copy of the TSD is lost together with the deleted segment. Integration into another chromosome will generate a balanced reciprocal translocation, or an unstable pair of dicentric/acentric chromosomes [not shown; (Yu et al. 2010b; Zhang et al. 2009)]

Our strategy for the production of genome rearrangements is shown in Fig. 2. The *pRAc* construct containing the *Ac* transposase gene and a pair of reversed *Ac/Ds* termini is introduced into rice lines via Agrobacterium-mediated T-DNA transformation. The expressed *Ac* transposase can excise the reversed *Ac/Ds* termini, leading to the loss of the DNA fragment containing the GFP gene. The reversed *Ac/Ds* termini form an active transposon and integrate elsewhere in the genome, with a preference for linked insertion sites. Integration into the flanking DNA will generate either a deletion or an inversion, depending on the orientation in which the *Ac/Ds* termini integrate. Insertion into another chromosome would generate a reciprocal translocation (not shown).

The *pRAc* construct was transformed into *Oryza sativa* ssp. *Japonica* cv. *Nipponbare* by Agrobacterium-mediated transformation. To facilitate downstream analysis, we screened 17 independent T-DNA lines by Southern blot hybridization to estimate transgene copy number. Three lines (Numbers 6, 7, and 9), containing a single-copy T-DNA insertion, were selected for starter lines. The rice genomic sequences flanking the T-DNA insertions in these three lines were cloned by inverse-PCR, and the insertion loci were confirmed by re-amplification of the expected fragment using primers specific for the T-DNA and flanking DNA. The T-DNA insertions in lines 6, 7, and 9 are located on rice chromosomes 1, 5, and 3, respectively. Minor sequencial changes were detected at the T-DNA insertion junctions: No. 6 (20 bp deletion), No. 7 (917 bp deletion and 25 bp insertion) and No. 9 (19 bp deletion) (Table 1). These sequence changes were assumed to have occurred upon T-DNA integration.

**Table 1:** T-DNA insertion sites of transformed rice lines

Transgenic line	T-DNA flanking sequence and chromosome location
No. 6	TTATAACAAGTATGCTTTAT– – –ATACTAGGTACTGGTACTCC
	Chr01:20381147…20381127– – –20381106…20381087
No. 7	CGCTGCAAGGTCGCAGGTAGTTTGTTTACACCACAATAATTCAGT– – –TAGTACCAGGGTTGTATTGA
	Chr05:25379609…25379590– – –25378674…25378655
No. 9	GCGGTCTTCTCCCCGGCCGC– – –CACAAGTGCACAACTACACG
	Chr03:1931635…1931616– – –1931598…1931579

For each transgenic rice line, the upper line shows the 20 nt sequence flanking the T-DNA insertion site, and the dashed line represents the T-DNA in a right border to left border orientation. The lower line indicates the chromosome and sequence coordinates according to TIGR rice genome release 6.1. The underlined sequence in transgenic line No. 7 indicates a 25 bp insertion of unknown origin

### Frequent somatic alternative transposition events

The alternative *Ac/Ds* transposition model predicts that the ends of the ITS located between the reverse-oriented *Ac/Ds* 5′ and 3′ termini will join together and form a 3.1 kb circular DNA containing the *Ubi*::*GFP* sequences (Fig. 2). Although the predicted circular DNA would likely be a transient product, indirect evidence for circle formation stems from isolation of alleles containing permutations of a 13 kb ITS in maize (Zhang and Peterson 2004). Here, a PCR assay was performed to detect circle junctions formed by ligation of the transposon-flanking sequences. A PCR primer pair (primer 1 and primer 2; Supplementary dataset 1) flanking the *Ac*/*Ds* termini can prime a PCR reaction only from a circularized DNA molecule (Supplementary Fig. 1). Using genomic DNA as PCR template, we did detect bands of the expected size in all three transgenic lines (data not shown). The bands were excised from the gel and directly sequenced using primer 1. Representative DNA sequence traces are shown in Supplementary Fig. 1. The first 19 nucleotides (nt) match the sequence flanking the *Ds* 5′ terminus. Exactly after nucleotide 20, all three sequence traces exhibit multiple peaks, suggesting that the PCR products contain a mixture of “footprints” derived from multiple independent somatic excision events. In contrast, the two lower sequence traces do not have multiple peaks; each sequence contains a distinct presumptive transposon “footprint”. In each case, the first 19 nt match the sequence flanking the *Ds* 5′ terminus followed by two non-matching base pairs and 19 nt matching the sequence flanking the *Ds* 3′ terminus. These latter two clear sequences most likely represent PCR products containing a single predominant *Ac/Ds* excision clone. These results support the hypothesis that excision of reversed *Ac/Ds* termini generates a circular molecule from the ITS. We also conclude that alternative transposition events can occur frequently in these rice vegetative tissues.

### Multiple putative germinal rearrangement lines obtained by marker-assisted screening and PCR analysis

The protocol for screening for transposition-induced rearrangements is shown in Supplementary Fig. 2. Seeds harvested from the three starter transgenic lines (T1 plants) were germinated on 1/2 MS plates containing hygromycin. The hygromycin-resistant (HPH^+^) seedlings (T2) were classified according to GFP expression: GFP^−^ seedlings (from 0 to 25 % in this class) were considered as putative rearrangement lines and analyzed further; the GFP^+^ seedlings were grown to produce T3 seeds which were in turn screened for HPH^+^, GFP^−^ plants. A few homozygous HPH^+^ GFP^+^ T2 plants from transgenic line No. 7 were identified by PCR analysis using flanking T-DNA primers. Seeds harvested from the homozygous T2 plants were directly screened for GFP^−^ plants without HPH screening. Genomic DNA was extracted from candidate rearrangement-containing seedlings. Three specific PCR primer pairs on the T-DNA region were used to test for rearrangements induced by the reversed *Ac/Ds* termini structure (Fig. 1). PCR pair A detects the CaMV35S promoter/*Ac* transposase junction, PCR pair B detects the *Ds* 5′ end/GFP junction, and PCR pair C detects the HPH gene. Based on the PCR patterns obtained, putative rearrangements could be classified as inversion or translocation (+ − + pattern) or flanking deletion (− − + or + − − patterns). The (+ − +) pattern could also be generated by fusion of the reversed *Ac/Ds* termini, i.e., deletion of the ITS without transposition of the *Ac/Ds* termini. Most rearrangement events exhibited (+ − +) patterns and thus appeared to be chromosome inversions or fused reversed *Ac*/*Ds* termini; approximately ten plants appeared to contain deletions. Several plants from starter line No. 9 yield approximately 10 % GFP^−^ and HPH^+^ progenies; however, in these cases all three PCR primer pairs yielded positive results, and their progenies were positive for GFP expression in the next generation. We conclude that the GFP gene in those plants was probably transiently silenced.

The alternative transposition system is expected to generate rearrangements containing one fixed breakpoint at the T-DNA insertion site and a second variable breakpoint ligated to one of the reversed *Ac*/*Ds* termini (Fig. 2). All the breakpoint sequences of the rearrangements described here were cloned by inverse-PCR using *Ds* specific primers (Supplementary dataset 1). A total of 25 independent events were identified from the three starter lines. By comparing the original T-DNA insertion sites (Table 1) with the cloned breakpoint sequences (Supplementary dataset 2), the rearrangements were classified as putative deletions, inversions, or translocations (Table 2).

**Table 2:** Rearrangement lines generated by alternative transposition events

Parental line	Rearrangement line	Rearrangement type	Rearrangement size (kb)^a^	TSD^b^
No. 6	M6-1	T1-7 Translocation		Confirmed
	M6-2	Deletion	0.34 (on construct)	NA
	M6-3	Deletion	72	NA
	M6-4	Inversion	100	NT
	M6-5	Inversion	2.5 (on construct)	NT
	M6-6	Inversion	900	Confirmed
	M6-7	Fused *Ac/Ds* 5′ and 3′ end	(on construct)	NA
No. 7	M7-1	Deletion	4.0 (on construct)	NA
	M7-2	Inversion	1200	Confirmed
	M7-3	Inversion	3.5 (on construct)	Confirmed
	M7-4	Inversion	53	Confirmed
	M7-5	Inversion	1000	Confirmed
	M7-6	Inversion	170	NT
	M7-7	Inversion	26	Confirmed
	M7-8	Inversion	9	NT
	M7-9	Inversion	1500	Confirmed
	M7-10	Inversion	820	Confirmed
	M7-11	Deletion	5.9 (on construct)	NA
	M7-12	Inversion	5.6 (on construct)	Confirmed
	M7-13	Deletion	0.34 (on construct)	NA
	M7-14	Fused *Ac/Ds* 5′ and 3′ end	(on construct)	NA
	M7-15	Deletion	5.6 (on construct)	NA
No. 9	M9-1	Inversion	87	Confirmed
	M9-2	Fused Ds 5′ and 3′ end	(on construct)	NA
	M9-3	Deletion	79	NA

^a^Rearrangement size indicates the size of the affected genomic segment; the T-DNA region is not taken into account except for those cases marked “on construct”, in which the rearrangement occurred entirely within the transgene sequences

^b^“TSD confirmed” indicates the presence of an 8 bp target site duplication flanking the *Ac/Ds* 5′ and 3′ termini in the indicated inversions and translocation. *NT* not tested, only one flanking sequence was cloned; *NA* not applicable, because TSDs are absent in deletions and fused end events

### Characterization of rearrangements derived from transgenic line No. 6

Seven independent rearrangement mutant alleles were produced from parental line No. 6, which has the T-DNA inserted on chromosome 1 (Table 2). The sequences of the junction fragments obtained by PCR indicate that the rearrangements derived from line No. 6 include one translocation, two deletions, three inversions, and one fused-end. For line M6-1 (translocation), both sequences flanking the *Ac/Ds* 5′ and 3′ termini mapped to a single site on rice chromosome 7 and contain the same 8 bp target site duplication (TSD). These results indicate that M6-1 represents a T1-7 reciprocal translocation generated by alternative transposition. Recently we reported the isolation of 17 reciprocal chromosome translocations induced by reversed *Ac/Ds* termini transposition in maize (Zhang et al. 2009). The translocation observed in M6-1 indicates that major chromosome rearrangements can also be induced by *Ac/Ds* alternative transposition in rice. Unfortunately, the M6-1 line failed to produce any seed, precluding further analysis. The two deletions included one contained within the construct, and a second extending 72 kb into the flanking rice genomic DNA. The three inversions range in size from 2.5 kb (within the construct) to 900 kb; in the latter, the presence of an 8 bp TSD at both inversion breakpoints was confirmed. Finally, the single fused-end event has the 5′ and 3′ *Ds* termini ligated together with concomitant loss of the ITS containing the *Ubi*::*GFP* sequence. Similar examples of fused *Ds* termini resulting from reversed-ends *Ac/Ds* transposition reactions in Arabidopsis were described recently (Krishnaswamy et al. 2010).

### Derivatives of transgenic line No. 7

**Fig. 3:**
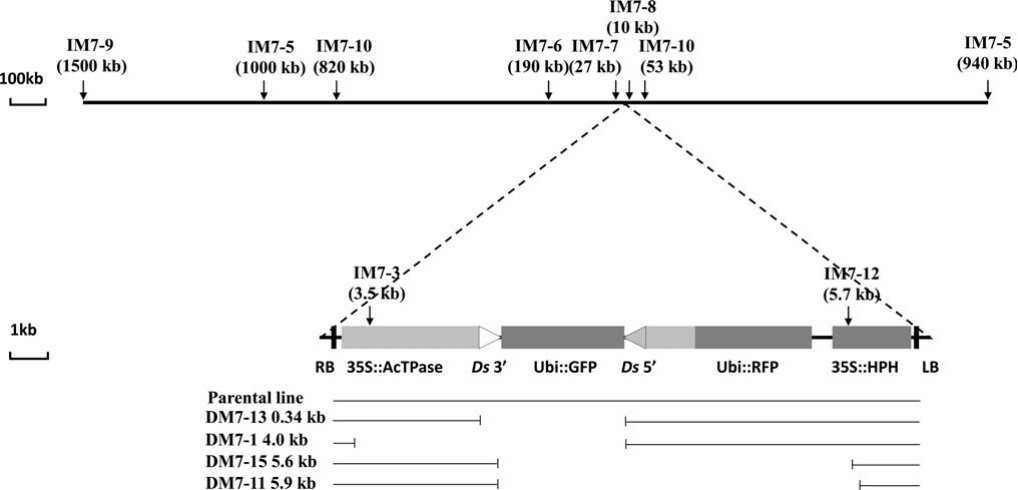
Map of inversion and deletion alleles derived from transgene line No. 7. *Upper*, map showing a portion of rice chromosome 5. The site of insertion of the transgene is indicated by the *dashed lines*. *Vertical arrows* indicate the breakpoints of each inversion; sizes are given in *parentheses*. Inversion alleles are indicated by prefix “I”. *Lower*, map of the transgene insertion. *Lines below the map* indicate the approximate breakpoints identified in each deletion allele. Deletion alleles are indicated by a prefix “D”. Inversion sizes do not include the T-DNA sequence, unless the breakpoint is within the T-DNA region

**Fig. 4:**
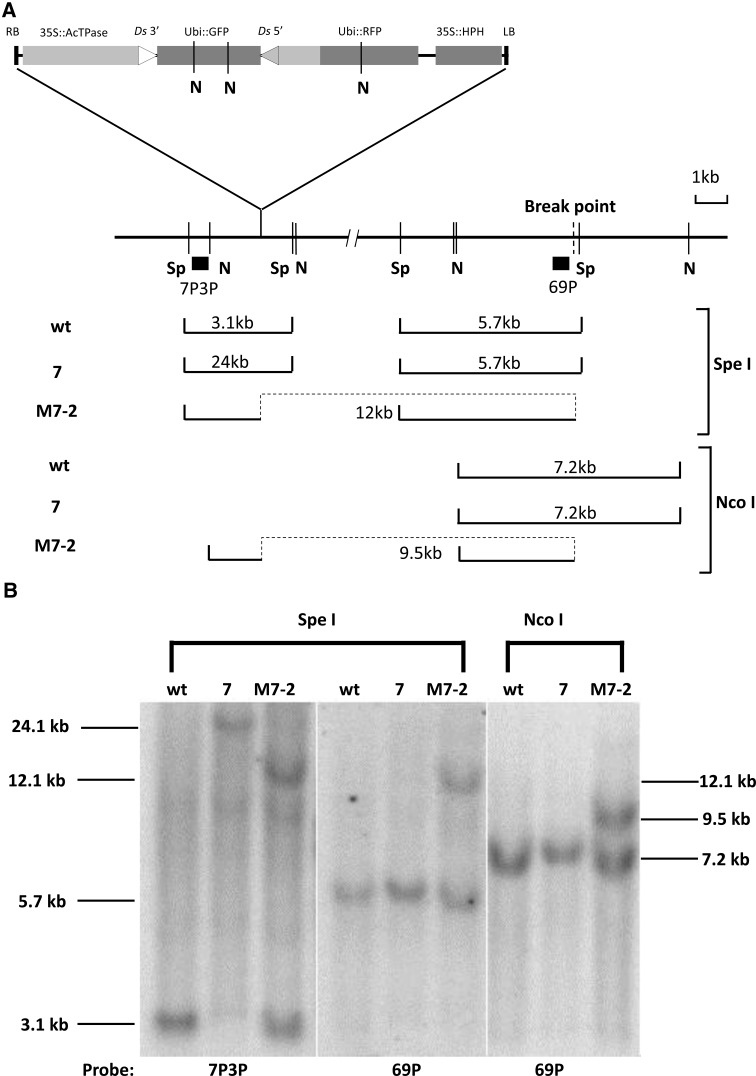
Southern blot analysis of inversion line M7-2. **a** M7-2 structure and restriction map. *Sp* *Spe*I, *N* *Nco*I. *Lines below the map* indicate the restriction fragments produced by digestion with *Spe*I or *Nco*I and hybridizing with the indicated probes marked with *black bars*. *Dashed lines* represent the two fragments joined together by the approximately 1.2 Mb chromosome inversion in M7-2. The T-DNA region is marked as in Fig. 1. The detailed structure of M7-2 is shown in Supplementary Fig. 3. **b** Southern blot analysis of plants containing wild type (Wt), parental No. 7 (homozygous), and M7-2 (heterozygous with wild type) chromosomes. Genomic DNA was cut with *Spe*I and *Nco*I; the *Spe*I blot was first hybridized with probe 7P3P and then rehybridized with probe 69. The M7-2-specific 12 kb *Spe*I bands hybridize with both 7P3P and 69 probes, as predicted

From parental line No. 7 (with T-DNA inserted on chromosome 5), we obtained four deletions, ten inversions, and one fused-end event. All four deletions and two of the ten inversions had breakpoints within the T-DNA region (Table 2). These results support the idea that reversed *Ac/Ds* termini transpositions exhibit local insertion preference, as for standard *Ac/Ds* transposition. In addition, small deletions may have been enriched in our screen due to two factors: (1) deletions beyond the right T-DNA border would remove the hygromycin-resistance gene and thus would be selected against during screening and (2) possibly, one or more gametophyte-essential genes may be located near the T-DNA borders. The inversions range in size from less than 5 kb (i.e., contained within the T-DNA region) to approximately 1.5 Mb (Table 2; Fig. 3). A subset of the inversion lines was analyzed by genomic Southern blot; the results from line M7-2, which carries a nearly 1.2 Mb inversion, are shown in Fig. 4 and Supplementary Fig. 3. The heterozygous M7-2 genomic DNA was cut with *Spe*I, and the blot was hybridized with flanking T-DNA insertion site probe 7P3P. A novel 12 kb band was observed in the M7-2 sample compared with the wild type plant. Because the T-DNA region does not have an *Spe*I restriction site, the homozygous parent line No. 7 exhibits a 24 kb band. The same blot was rehybridized with probe 69P, which originates from a site near the breakpoint, approximately 1.2 MB from the T-DNA locus. The same 12 kb band is detected with both 7P3P and 69P probes, indicating that these sequences are now conjoined as a result of the 1.2 Mb inversion. In addition, results from an *Nco*I digest probed with 69P also show an extra band in the M7-2 sample, again supporting the presence of a 1.2 Mb chromosome inversion.

### Derivatives of transgenic line No. 9

**Fig. 5:**
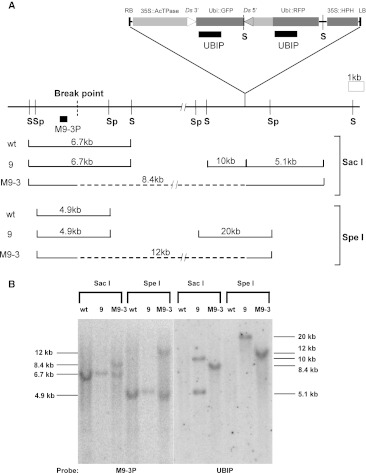
Southern blot analysis of deletion line M9-3. **a** M9-3 structure and restriction map. *S* *Sac*I, *Sp* *Spe*I. *Lines below the map* indicate the restriction fragments produced by digestion with *Sac*I or *Spe*I and hybridizing with the indicated probes marked with *black bars*. *Dashed lines* indicate sequences deleted as part of the 79 kb deletion in M9-3, resulting in predicted bands of 8.4 kb (*Sac*I digestion, probe M9-3P) and 12 kb (*Spe*I digestion, probe UBIP). The T-DNA region is marked as in Fig. 1. **b** Southern blot analysis of plants containing wild type (Wt), parental No. 9 (hemizygous), and rearrangement M9-3 (heterozygous with wild type) chromosomes. Genomic DNA was cut with *Sac*I and *Spe*I; the blot was first hybridized with M9-3P probe and then rehybridized with UBIP probe. The M9-3-specific 8.4 kb *Sac*I and 12 kb *Spe*I bands hybridize with both M9-3P (*left*) and UBIP (*right*), as predicted

Transgenic line No. 9 (with T-DNA inserted on rice chromosome 3) gave rise to rearrangements M9-1, M9-2, and M9-3. Analysis of the cloned breakpoint sequences indicates that M9-1 contains an inversion in which the *Ac*/*Ds* 5′ terminus is joined with the rice genomic sequence 87 kb upstream of the left T-DNA border, and the *Ac*/*Ds* 3′ end is joined to the sequence immediately downstream of that site. Both junctions contain an identical 8 bp TSD, thus confirming that this inversion was generated by a single transposition event. A single plant from No. 9 gave rise to 19 GFP-negative seedlings. Sequence analysis of two of these seedlings indicates that they contain identical structures in which the 5′ and 3′ *Ac*/*Ds* termini are joined together. The remaining 17 GFP-negative seedlings were analyzed by PCR using *Ac/Ds* 5′ and 3′ end-specific primers; the results suggest that all contain the same fused *Ac*/*Ds* termini structure. Most likely all of these 19 seedlings were generated in a single pre-meiotic event in which the *Ac/Ds* 5′ and 3′ termini fused together (Krishnaswamy et al. 2010). In M9-3, the *Ac/Ds* 5′ end is joined with a site 79 kb from the right T-DNA border, suggesting that M9-3 has a 79 kb flanking deletion. To test this possibility, we performed Southern blot analysis on a plant heterozygous for the M9-3 allele. Genomic DNA was cut with restriction enzymes *Sac*I or *Spe*I, and hybridized with a probe (M9-3P) from the breakpoint, approximately 80 kb from the T-DNA insertion site (Fig. 5A). Novel bands of 8.4 kb (*Sac*I) and 12 kb (*Spe*I) were found in the M9-3 line compared with wild type and parent No. 9 (Fig. 5B). Reprobing of the same blot with a probe (UBIP) from within the construct produced 10 and 5.1 kb *Sac*I bands in parental No. 9, and an 8.4 kb *Sac*I band in M9-3 lane; with *Spe*I, parent 9 hybridizes with a 20 kb band while M9-3 gives a 12 kb band. Importantly, the 8.4 kb *Sac*I and 12 kb *Spe*I bands detected in M9-3 hybridize with both the M9-3P and UBIP probes. These results, together with the sequence data indicating that the *Ac*/*Ds* 5′ terminus is joined to the M9-3P-containing fragment, indicate that M9-3 contains a 79 kb deletion. Although the deletion structure was successfully transmitted to the next generation, we did not detect any plants homozygous for the deletion among 26 plants tested by PCR. This 79 kb deleted region contains approximately 16 predicted genes (Plant Genome Database; http://www.plantgdb.org). Except for two genes encoding putative transposon proteins and three genes encoding glutathione *S*-transferase, the other genes are of unknown function. Possibly one or more of these genes are essential for plant viability.

**Fig. 6:**
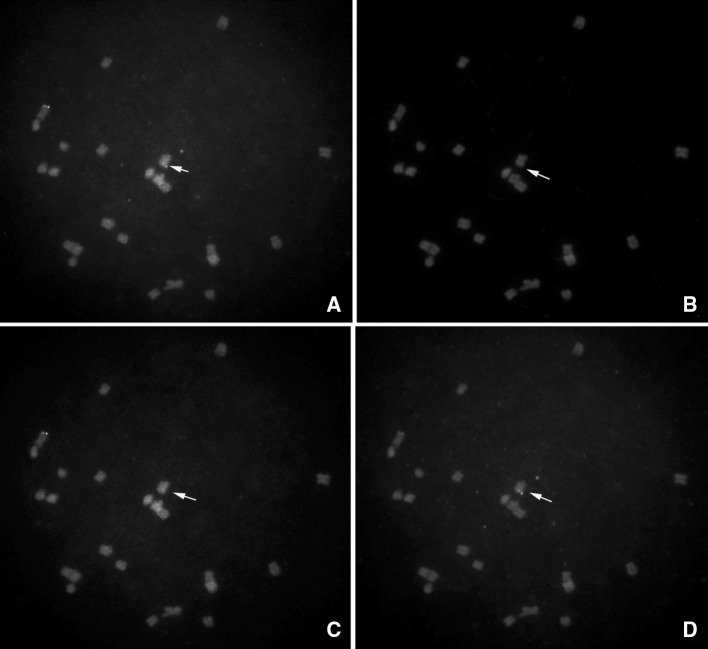
FISH analysis of M9-3 allele. Cells of plants heterozygous for a rearrangement and corresponding normal chromosomes were characterized by FISH of mitotic metaphase chromosomes using probes 9D and 9F. The probe 9D (*green*) is located within the 79 kb deleted region. The probe 9F (*red*) is located in the flanking region. *Arrows* indicate the chromosome with the 79 kb deletion. **a** The 9D and 9F signals are shown together; **b** only DAPI; **c** only 9D; **d** only 9F (color figure online)

To further test whether the internal 79 kb region on the M9-3 allele is deleted, we performed FISH analysis on rice mitotic chromosomes. FISH probe 9D is composed of two DNA fragments (3.7 and 3.8 kb) derived from the 79 kb deleted region. FISH probe 9F is a positive control composed of two DNA fragments (4.7 and 3.3 kb) located nearby the deleted region. The FISH results are shown in Fig. 6. In addition to a normal chromosome 3 with both 9D and 9F signals, one chromosome (presumptive deletion) contains only the 9F signal. These results confirm the presence of the deletion in the M9-3 allele.

## Discussion

Recently Xuan et al. (2011) have shown that a pair of linked *Ds* elements at the rice *OsRLG5* locus can also undergo alternative transposition events to generate a variety of chromosomal rearrangements. Here, we show that it is possible to generate similar rearrangements at any rice locus by integration of a transgene construct containing relatively short (~250 bp) terminal segments of the *Ac/Ds* transposable elements. Lines containing the transgene construct on a T-DNA vector integrated at three different loci produced a variety of chromosome rearrangements including inversions, deletions, and one translocation. These events establish the proof of principle of this approach, i.e., that multiple rearrangement events can be generated from each transgene insertion and that these rearrangements fall into classes that are predicted by the *Ac/Ds* alternative transposition mechanism. This method may be generally useful for functional genomics and chromosome engineering.

### Frequency of alternative transposition-induced rearrangements

Previously, researchers have reported wide variations in the frequency of *Ac/Ds* transposition in rice, ranging from 0.1 % of F2 seeds containing germinal *Ds* excisions (Kolesnik et al. 2004) to greater than 70 % transposition in a system using callus-derived regenerated plants (Kim et al. 2004). Our system utilizes an *Ac/Ds* construct that is modified from a vector used for activation tagging in rice (Qu et al. 2008). In the activation tagging system, Qu et al. (2008) reported transposition frequencies of 43.4 % in T2 seeds. In later generations, the germinal transposition frequency varied depending on transgenic line: one-third of the single-locus T-DNA transformants showed high transposition frequencies (20–83.3 % of plants having at least one transposition per plant). In our material, screening of T2 plants for GFP^−^, HPH^+^ plants yielded transposition frequencies ranging from 0 to 31 % with marked variation in different plants. Over all, the frequency of GFP^−^, HPH^+^ seedlings was approximately 10 % among all seedlings tested from parent plants heterozygous for the transgene locus. This frequency is much higher than that reported for a non-tissue-cultured *Ac*–*Ds* transposon tagging system (0.1 %) (Kolesnik et al. 2004). The low frequency reported by Kolesnik et al. (2004) may be due in part to selection for unlinked transposition events, which will reduce the total number of recovered events due to the local transposition preference of *Ac/Ds* (Athma et al. 1992; Dooner and Belachew 1989; Yu et al. 2011). In our materials, transposition frequency appeared to decline as more advanced generations were tested. Alternative transposition events could be detected in all of the three original T1 lines. In the T2 generation, about 60 % of the plants still had detectable germinal transposition events. Somatic transposition still occurred in T3 and T4 plants based on the detection of ITS circle formation by PCR; most plants tested showed the presence of the somatic circle junction, but the band intensity in the T3 and T4 plants was significantly weaker than for T1 plants (Supplementary Fig. 4). Together, these results are consistent with reports that callus regeneration significantly enhances *Ac/Ds* transposition (Greco et al. 2003; Ki et al. 2002; Kim et al. 2004).

As shown in Table 2, the 25 rearrangements described here include 14 inversions, 7 deletions, 3 fused-ends, and 1 translocation. It is noteworthy that inversions exceed deletions by twofold. The accepted models of alternative transposition predict that inversions and deletions should be formed at equal frequencies because they are alternative outcomes of the same type of reversed-ends transposition reaction. The most likely explanation for the apparent deficiency of deletions is that larger deletions are not transmitted due to the loss of one or more genes which are essential for the function of the gametophyte. This explanation is supported by the fact that the largest deletion obtained here is 72 kb, whereas we obtained seven inversions of 100 kb or larger.

### Fused *Ac*/*Ds* ends structure produced by abortive transposition

The maize *Ac/Ds* transposable elements are thought to transpose via a cut-and-paste mechanism. Previously, Levy and coworkers detected extrachromosomal *Ds* circles in maize and transgenic tobacco somatic cells. Because they did not observe integration of the circular *Ds* into the plant genome, they concluded that the circular *Ds* is likely an abortive transposition product (Gorbunova and Levy 1997). Small extrachromosomal circular DNA molecules would lack a centromere and hence would not be stable in the plant genome. In our study using reversed *Ac/Ds* termini, abortive transposition events would result in deletion of GFP and fusion of the *Ac*/*Ds* termini. We obtained a number of these “fused ends” events, the sequences of which are shown in Table 3. Fused-end sequences 1 and 2 contain insertions of 5 and 3 nt, respectively, between the intact *Ac*/*Ds* ends. Sequence 3 has, in addition to a 3 nt insertion, a 3 nt deletion from the 3′ end of *Ac*/*Ds*. The inserted nucleotides are different from those originally present at the *Ac*/*Ds* 5′ or 3′ flanking sequences. These results suggest that ligation of the *Ac*/*Ds* termini to produce fused ends probably occurs via the non-homologous end-joining (NHEJ) pathway.

**Table 3:**
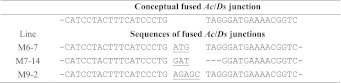
Sequences of fused *Ac/Ds* end junctions

Sequences indicate a conceptual precise fusion of *Ac*/*Ds* 5′ and 3′ termini (upper entry), and the sequences of the three fused ends events obtained in this study. Underlined sequences indicate insertions of unknown origin; dashed lines indicate deleted nucleotides

### Use of alternative transposition for mutagenesis and chromosome manipulation

Loss of gene function is a powerful tool for genetic analysis. Targeted gene replacement by homologous recombination is highly efficient in yeast (Winzeler et al. 1999). Site-specific gene disruption has been developed for mice, but is difficult or unavailable for most other vertebrates, including rats (Koller and Smithies 1992; Rossant 2003). In plants, methods for directed gene modification are still inefficient (Townsend et al. 2009), and hence most functional genomics approaches rely on collections of knockout mutations. Arabidopsis, rice and maize are the three most commonly used model plants. Because of its smaller genome size and simpler genome composition, Arabidopsis has been the subject of numerous insertional mutagenesis projects, resulting in collections containing approximately 379,674 independent insertion events targeting 91 % of the predicted genes (http://signal.salk.edu/Source/AtTOME_Data_Source.html). In contrast, only about 200,000 T-DNA or transposon insertion lines, which together mutate about 50 % of the predicted genes, are available in rice (Krishnan et al. 2009). Achieving saturation mutagenesis of the maize genome is even more challenging: it is estimated that 1,800,000 independent insertions would be required to tag every gene in maize with a 95 % probability (assuming completely random insertions, a 2400 MB genome size, and 4 kb average gene size) (Haberer et al. 2005; Krysan et al. 1999). In contrast, approaches based on the alternative transposition can remove ten or more genes in a single transposition event as in the rice M9-2 deletion reported here and the 4.6 cM deletion flanking the maize *P1* locus (Zhang and Peterson 2005). In addition, deletions would also remove gene regulatory elements, which may be otherwise difficult to detect by simple insertion mutations. Finally, alternative transposition events generate other structural rearrangements such as chromosomal inversions and translocations, which can also be a valuable resource for genetic research (Maguire 1972; McClintock 1931). Compared to the natural translocation or inversion lines, the large rearrangement lines generated by alternative transposition contain defined endpoints and can be used to manipulate copy number of defined chromosome segments (Birchler 1980; Birchler and Levin 1991; Yu et al. 2010a; Zhang et al. 2009).

Although our system utilizes the *Ac/Ds* system, it seems reasonable to propose that many “cut and paste” transposons may also undergo alternative transposition. For example, Drosophila *P* elements undergo alternative transposition events termed Hybrid Element Insertion (Gray et al. 1996; Preston et al. 1996). For a number of reasons, alternative transposition may actually be more applicable for research in animal versus plant systems. First, due to differences in gamete development, large deletions are more likely to be transmitted in animals. Development of the gametophyte stage of the plant life cycle involves several post-meiotic haploidic mitotic cell divisions (Candela and Hake 2008). Multiple, widely-distributed genes are expected to be essential for completion of these mitotic divisions and survival of the gametophyte. Gametes containing large deletions will likely have severely reduced transmission frequency. In contrast, animals do not have such mitotic cell division processes; the products of meiosis develop directly into gametes. Thus, sperm and egg cells containing large chromosome segmental deletions or even chromosome arm losses are still functional in fertilization in animals including human (Maranda et al. 2006; Massa et al. 1992). Second, major chromosomal aberrations including deletions, duplications, inversions, and translocations are frequently associated with human congenital diseases and cancer (Albertson et al. 2003; Rabbitts 1994). Thus, we propose that alternative transposition may be used as both a functional genomics research tool and for the development of model disease systems for medical research.

## Electronic supplementary material

Below is the link to the electronic supplementary material.

Supplementary material 1 (PDF 1512 kb)
