# Supplementary material for: A transgenic system for generation of transposon Ac/Ds-induced chromosome rearrangements in rice
Source: Theor Appl Genet. 2012 Jul 14;125(7):1449–62. doi: 10.1007/s00122-012-1925-4 (PMC3470690; doi:10.1007/s00122-012-1925-4)
Supplement: Supplementary file 1 — Supplementary material 1 (PDF 1512 kb) [file 122_2012_1925_MOESM1_ESM.pdf]

Supplementary Figure 1

Sequences of *Ds* 3' and 5' junctions in putative excised circles.

Top diagram shows the 20 bp sequence of the Inter Transposon Segment (ITS) flanking the reversed *Ds* 5' and 3' ends. The products were obtained by PCR amplification of transgenic genomic DNA using primers 1 and primer 2. Sequence traces show the results of sequencing using primer 1. Samples a, b and c exhibit considerable sequence heterogeneity downstream of the footprint site; whereas samples c and d give clear sequences. Lower lines show the sequences of samples c and d; the transposition footprint is shown in small letters.

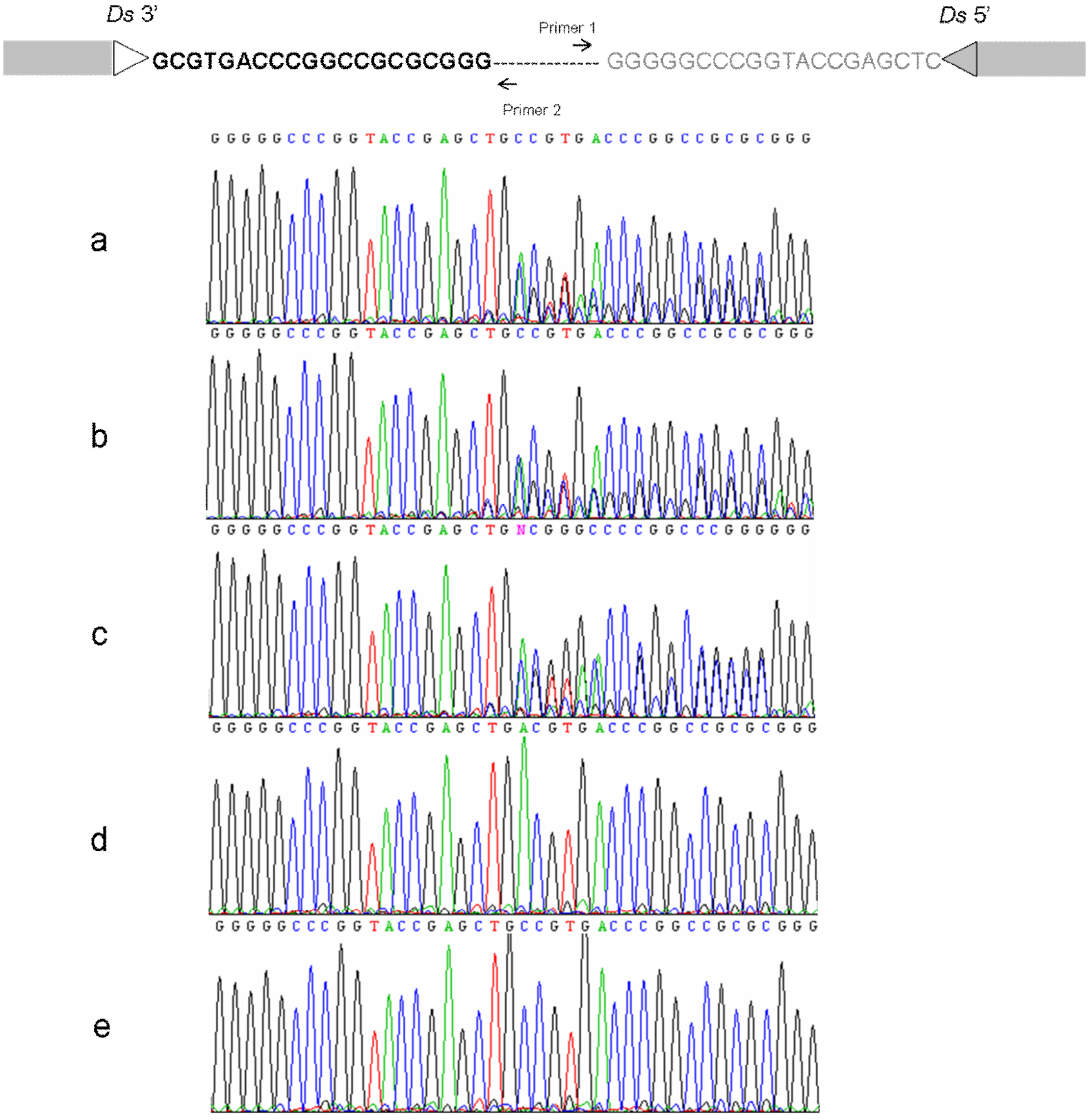

Transposition footprints

d GGGGGCCCGGTACCGAGCTgaCGTGACCCGGCCGCGCGGG

e GGGGGCCCGGTACCGAGCTgcCGTGACCCGGCCGCGCGGG

Supplementary Figure 2

Screening and selection procedure for rice rearrangement lines.

Single copy T1 plant lines were identified by Southern blot. The homozygote T2 plants were identified by PCR analysis with flanking T-DNA insertion site primers. Seeds harvested from T1 or T2 plants were germinated and screened by GFP or Hygromycin resistance to verify presence of the transgene. T2 plants homozygous for the transgene insertion were identified by PCR analysis using flanking T-DNA primers. The ⊗ symbol indicates self-pollination.

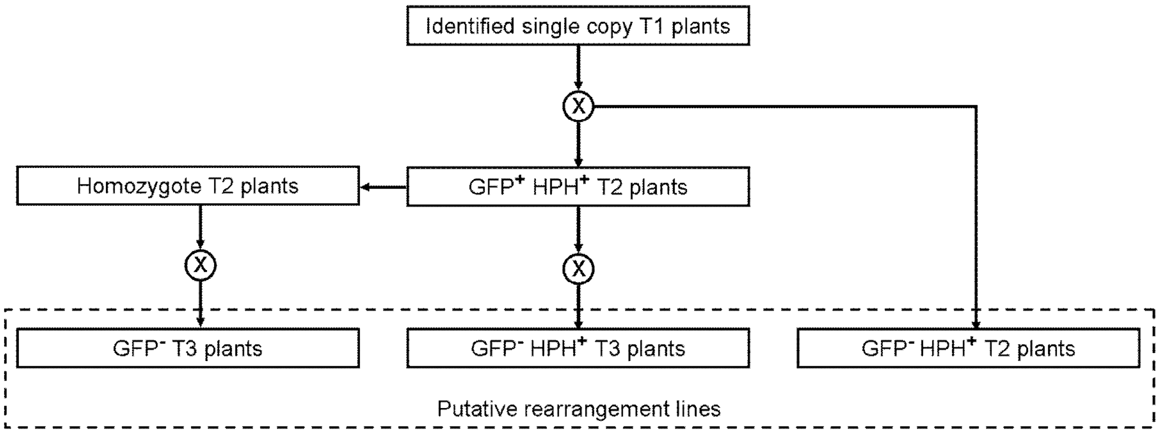

Supplementary Figure 3

Detailed structure of M7-2 allele.

The symbols for restriction map and probe are the same as shown in Figures 1 and 4.

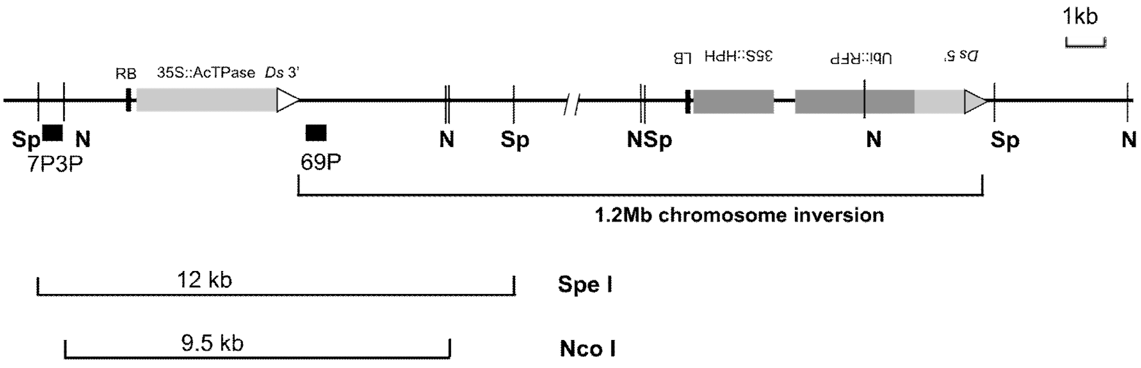

Supplementary Figure 4

Alternative transposition frequency declines from T1 to T4 generation plants. The panel shows the results of PCR-based detection of excised circle junctions in genomic DNA from T1 and T4 generation plants. PCR was performed using primers 1 and 2 as shown in Supplementary Figure 1. T1 plants No. 6, 7 and 9 gave strong bands, indicating frequent somatic alternative transposition events. Control lane contains genomic DNA from a GFP-negative plant which lacks the intact transgene structure and does not amplify a circle junction fragment. The five T4 plants shown here are progeny derived from T1 plant No. 7. T4 plants 1 and 2 are negative; T4 plants 3, 4 and 5 gave weak bands.

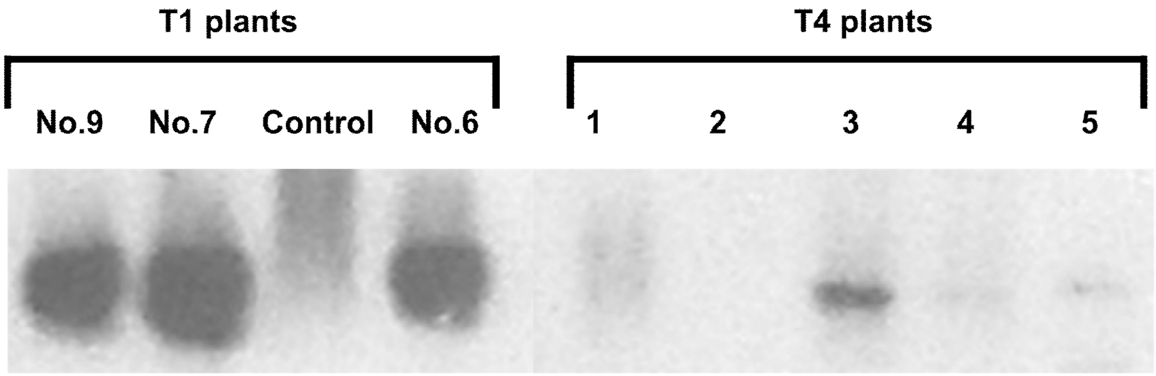

Supplementary dataset 1. Oligonucleotide DNA primers

For detection of putative somatic excision circles:
Primer1: 5′-CTGGAGTTCGTGACCGCC-3′.
Primer2: 5′-AGAGAGGGGCACGACCG-3′.

For preparation of Southern blot hybridization probes:
7P3P probe: 5′-GCAACCGTTGTCATGTTTCCCT-3′ and 5′-CGATGGGGAGGTCAGGGG-3′.
69P probe: 5′-GCAGGTGTGGCAATGGCA-3′ and 5′-TCGGTCAGTCAGTCGGTGGA-3′.
UBIP probe: 5′-CGACGAGTCTAACGGACACC-3′ and 5′-CGTATGAAGGCAGGGCTAAA-3′.
M9-3P probe: 5′-GGCGGCGGCGGCGACG-3′ and 5′-CGTCGCCGCCGCCGCC-3′.

For diagnostic PCR assays:
Ar: 5′-GGTTGTGGCTGTGGTTGTGGTTCTGGTT-3′.
Af: 5′-AGATAGTGGAAAAGGAAGGTGGC-3′.
Bf: 5′-CTGGAGTTCGTGACCGCC-3′.
Br: 5′-CCCGTTTCCGTTCCGTTTTCGT-3′.
Cf: 5′-GCGAAGAATCTCGTGCTTTC-3′.
Cr: 5′-CTGGGAACTACTCACACATTATTATGG-3′.
.
For isolation of T-DNA insertion sites or rearrangement breakpoint sequences:
LB1: 5′-GCATATAAGAAACCCTTAGTATGTATTTGT-3′.
LB2: 5′-TCAACACATGAGCGAAACCCT-3′.
LB3: 5′-GTACTAAAATCCAGATCCCCCG-3′.
LB4: 5′-CTGGGAACTACTCACACATTATTATGG-3′.
RB1: 5′-TGGCGAAAGGGGGATGTG-3′.
RB2: 5′-TGTCGTTTCCCGCCTTCAGT-3′.
RB3: 5′-CGCCAGGGTTTTCCCAGTCAC-3′.
RB4: 5′-ACCCGCCAATATATCCTGTCAAAC-3′.
Ds5’f: 5′-TTCCCGACCGTTTCACCG-3′.
Ds5’r1: 5′-GCTCTACCGTTTCCGTTTCCGTTTACCG-3′.
Ds5’r2: 5′-CCCGTTTCCGTTCCGTTTTCGT-3′.
Ds3’r: 5′-TTGCGGGACGGAAACGAAAACGGGATA-3′.
Ds3’f1: 5′-ATGAAAATGAAAACGGTAGAGG-3′.
Ds3’f2: 5′-GTAGATGTATTTTACCGACCGTTA-3′.

For preparation of FISH probes:
9D-3.7kb: 5′-ATCGTCATCATTCTTCCTCCCAT-3′ and 5′-GCTTCTTGTCTCCGTGTCCCG-3′;
5′-CTCGTCTCCTCCTCCGCCA-3′ and 5′-CCCAGATAATGAGACCCACCACT-3′;
9D-3.8kb: 5′-GTAGAGGAGAGGATAAATGTTGTGGTG-3′ and 5′-TGAGGGAAGGGGGAAGGGAC-3′;

5′-GTCCCTTCCCCCTTCCCTCA-3′ and 5′-CCCTACTCGTCCAAGGTATCTCTACTCT-3′.

9F-4.7kb: 5′-CGTTTTCACCCCTACTCAATGG-3′ and 5′-TTTTGTCCGCTTCCCTTTTTTC-3′;
5′-GAAAAAAGGGAAGCGGACAAAA-3′ and 5′-TAGCATAACAAAAGAAAAGGGTGGT-3′;
9F-3.3kb: 5′-CTGTGGCAGGGTAGTGATGATTTAG-3′ and 5′-TTTTGGGAGAATAAGGGGAGATAGG-3′;
5′-GGTGGCAGTGGAGTCTTGGTGA-3′ and 5′-GCAGGAGAGCGAGGGGC-3′.

Supplementary dataset 2. Cloned breakpoint sequences.

Yellow highlighted sequences indicate target site duplications (TSD) detected by sequencing both 5’ and 3’ *Ds* insertion junctions. For deletions only one *Ds* 5’ or 3’ end sequence is present; the underlined bases indicate the terminal nucleotide of the *Ds* 5’ (beginning of the sequence) or 3’ (end of the sequence) ends.

M6-1

TTGAAAATACAAACAAAAAAGGGGACGAATTTTGGCAAATTTTCTGACCATC
TCTCTGCAAGGTTGGGTTCGAGAGCATCGTGCATGTATGGCGCGCGACTCG
CCGG

M6-2(on construct)

CTTCGCTAACATAAGAAGCCATATAAGTCTACTAGCACACATGACACAATAT
AAAGTTTAAAACACATATTCATAATCACTTGCTCACATCTGGATCACTTAGCA
TGCATAAACTATTACAACCAAGGCTCATCTGTCAACAAACATAAGACACATT
GCTCATGGAGAGGAGCCACTTGCTACATCTTCATTATTCTTAGAAAATTCTAT
TGCGTCTTCATCCTGTTAATACACAAAAATAAGTCAGTTTTGGATAAATAAAT
ACATATAGAAGAACATGAATTGATATGCAGGGAGTATAAATAAATACATATAG
GAGAACATGAATCTGTGAACTAACACGGCTGGGAGCTAGGCAGCTAGCAGC
TAGCGC

M6-3

ATTATTCAGTCTCATGTGATATTCGCTAATGGATCACTTTTATCATATATACAC
CAATCTCTCCACCGTCATGGCCTTTTCTATTTCCATATTGTGATTGACTAGTG
TTGCAGACTACAGTTACACACATGAACACATAACACACACACAAAATAATTAT
AGGGCCTCTAAGAGGTCGGGGGATATTATTAGAGAGATAGGCTCAACGCGA
TAGTGTATTCATCACGGAGTATTACATATATAAACCACAACCCTCCATACAAT
TAATTAGTAAAATGAAGCATTTTTAATAGTCCAAGGGGTAAAGTGAGAAAAAA
TAGTTTAGGGGTTACGTGGTCTAATGTAATGAAATAGTTTATGGCAGTAAAA
TAGTTTTCCTTTTCAAAATATATATTCTCAAATCTCAATGAATTAAGCTATTCT
CTGTAATAAATAACTGAAGAGTACATAAGCGGAATAATGTGTACATGTCTCA
AGCACTACTGCACTAGCTCTGCCATGTGCCCATGTCTGGTCCCATCCAGAG
AAACTACCCTGCTGCTAGCTCATAAACACATTACACATAGGGAATACATTGC

ACCGTAAAAGTTCTCTGTATATAGAATCAACGGTGGATGCTAGGTGTGGTTC
AAATAAATAGTAAAGCTGAACAATAATGGACACGAACTGCGC

M6-4
TTCGCTAGAATAAGATATTACGATACAGCAGGCTAGGAATTAATATAATTGTA
TAGGCGTCAATTAAAATCCCAAGGTTGTTTTGCCCGGTTGAGTTCCCTTTTT
CTTAAGCTACATAGGTTGAGTAAATGTTTGTAGGGATGGAGTCAAAGGAAAG
TGGGTAACATCAAGGTGCATCTGACTATAGTAAGGGAATCCTTCACCGGAG
TGAGATTGCAAGGGGCTTGCGGGCCCTCTCTAGTGGGGAGGACTTGCTGA
GGATGAAGCTCAAGCTCCACTGTCTTTGGTCTGGCTTCTCTTGAGAGCACC
ATTGCTTGCTTACAGTCTCGAGTTGCCTATCTTCGTGATGGTGATGCCAATA
CTGCCTTTTCTACCAGTAGTCCCGTTATGGCAAGAAGAAGAATTTTATTGCT
AATCTCAGGGCGAGGGAATGAGGGATCTGGTAGTTACTAGCCAGGATGAGT
TGACAAGTTATCTATAGGATCTATATGGTGTTTGAATCTTCTAAAGATGAAGA
TAAAGATGAAGAGATGAAGATTAAGTGTTTCATGCAAAACGAGGTGGTAATA
GCGTGTGATTAATTAAGTTTTAATTGTTACAAACTTGGAAAATATATTAATATG
ATATTTTAGAATAACTTTCATATA

M6-5 (on construct)
ATCTCTGTCGCTGCCTCTGGACCCCTCTCGAGAGTTCCGCTCCACCGTTGG
ACTTGCTCCGCTGTCGGCATCCAGAAATTGCGTGGCGGAGCGGCAGACGT
GAGCCGGCACGGCAGGCGGCCTCCTCCTCCTCTCACGGCACCGGCAGCTA
CGGGGGATTCCTTTCCCACCGCTCCTTCGCTTTCCCTTCCTCGCCCGCCGT
AATAAATAGACACCCCCTCCACACCCTCTTTCCCCAACCTCGTGTTGTTCGG
AGCGC

M6-6
GCGCCAATCAGATGGCCTCCGGCAGTCCGGCGAAAGGGAGGTCGACCACC
TTGTCCAACTGTACTACGAGTGTTTCGTGTCCAATCGGGTGGCGATGCGAC
GATGATTATACCGCAAACTTACTTCACCATGTTTCCTGCCAAAAATTGACAG
CCATGATCTTCAAGTGACATACCAATTATTCCCAACCCAAATCGGGCATGCT
TTAATTTTCCAGCCAGAAAAGAAAACAATGCAAGTAATATGATGCACGAGAC
TGCTAGCTCCTCATTTCGTTTTATCTCCAGGATAAAATTGAATAAAATTCCAT
TTCATATAGGAGTATGAAACTATCACATACTCAAATGTTCGCACATTTCTTTT
TGTACATGACCAAAACATTTCACAATAACCACAGGAGATGGTTCTTCCTGTC
TTCTTCCAAACCCCAAAAGGAACAAGACTGAAAAATGCAAAACAATGCACAG
CTGTCTTACTCGCTCAGTGCACAAATTTGAAATACAGGATTACTAGGATCGC
CAACACAAGGAGTGCTATGATGAAACCTATGATCCACTTGTTCCTGTCCATC
CTCCTCACCATGGCCCCAATGATCCTTCTGCTCTTTCCGATGTTATCATCCA
CGTTATGCAACTGCAACACCAACAAAGATATGAATAACAGAGGGGGAAGAC
AAGGAAGCATGCATGGGCCTAAATCCATGTACATTAATCAAACATGATTTGT
TTATTTATTTTAACAATATAATGCTACTGTGATCTATATCATATTTGATTAACT
GCTGAACAATATAATGCTACTGTGATCTATATATCATATTGCCGATTTGTCGT
CGGTTGATTGTGCTTCAACTTCAATATCGACGAACAGTTATAAGCAGGTTCT
GGAGGCCTAGAAATTACCACATTAGGCGGAGAAAAAAAACTAAACTATATTA

M6-7 (fused ends on construct)
AAACTAACAAAATCGGTTATACGATAACGGTCGGTACGGGATTTTCCCATCC
TACTTTCATCCCTGATGTAGGGATGAAAACGGTCGGTAACGGTCGGTAAAAT
ACCTCTACCGTTTTCATTTTCATATTTAACTTGCGGGACGGAAACAAAA

M7-1 (on construct)
GACCTGCAGGCATGCAAGCTTGGCACTGGCCGTCGTTTTACAACGTCGTGA
CTGGGAAAACCCTGGCGTTACCCAACTTAATCGCCTTGCAGCACATCCCCC
TTTCGCCAGCTGGCGTAATAGCGAAGAGGCCCGCACCGATCGCCCTTCCC
AACAGTTGCGCAGCCTGAATGGCGAATGCTAGAGCAGCTTGAGCTTGGATC
AGATTGTCGTTTCCCGCCTTCAGTTTAAACTATCAGTGTACTGAATTATTGTG
GTGTAAACAAACTACCTGCGACCTTGCAGCGAGTAAAATTAAACCCATTCTC
AACACTTATTACTCTGCTCCCTCGGCCTTAAAATATAAATATTTTTAAAATAAT
ATAAATATATTAAGAAAGTATAT

M7-2
GAACCGTTTAGGATTACCTTACTGGTTTTAAGGGTTNCAGCACCGCTTGGTC
ACTGCATGAACAATGAAAAATCTTTAACAAAATTAATATGATTTTTGAAACCA
ACTTTGTTTCTGCAAAAAATACACGACAGTTTGAAAAACGCGTACTTGGAAA
AAGAGACTGTGGGAGTTAGAAAAGTACAGTGTACTGGAGAACACAGCCTAG
CTTCNNAATCTTATCCTTCCGTAATCCGTAGCTCTTCAGAATCCAATGGCTA
CAAGGAAGAGAGTTTCTCCCCCAAAGGTGAAACGGAAACAAAGTCCAGTCC
ACGGAAGTGCGTGTCACGCTATAATACCGACTCAAAACGATTCCCTTCTCCC
ATCCATCCAGCTACACCACGTACACTACCGTGCCCAACTCTGCAACCCAAA
GCTGAAGAGGCTGCACCTGCACAAAACCAAGCAAGCTGTGACGGCTCACG
AGCGCGTGACCGCGTGAGAGAGAAAAAAGAATTGAATACTAGTAGAAGACA
ATGGTTCGGTGGGATGGCCAGGTCGAGACCCGG

M7-3 (on construct)
CTTCTACTCGCAAAACAAATTCCGTATTCTCTGCATATGCTCAAGGTATATAT
TAGAAAAACAGTAGCAATAGCATTAGCATTACTAATTGGTTGTAGATTGGGA
AGCATCATATTGACTGTAGAATAATACGAAAAATCTGTTTATAACAGGGTTGA
AAAGAAAAGCTGAAGCCTCTTCTAGTCGGATTCAGAATGTACGTGCACGT

M7-4
AGACAAACCAAATCTGCCNAGCGAATGAGCATTCTAAATGGACCTTCAGCTA
GCAGCATCAGTGCGGTGATGAACACTGCCATTCTGATGGACCTTTGGCGAG
CAGCATCCGTGCGGTGAAAACTGGCCCTCCGTTTAGTTTTCTCCCTGCAGC
CTTCACGTAACCGACTGTAGCGAAATATTCTGTCGGCATTGCGTTTCCGCTT
TCGCGGTGAACTGGCCCGG

M7-5
TATCACCAACCAAGGGATGCTCCCGCAGCTCAAGGCCATGAGAGACGAGCT
GTTCAGAGGCCACTACGTCCTCGACACCTTCAGACACCGGGCTGATCTCCT
CCAGAAAGAGGAGGAGAAAGAAGATGAACAGGTGAGAAGCTCGTTTGCAAT
GTCAAGGCTCAATCCTGCCAAGCGAATCCGTTTCTCTCGTGCTAGAACTAGT

AGCTTTCAGGATTTGGAGTCCATGATTCGTAGCCTGGAAGACGCCATTGCT
GACACAAAGGAGTTCATCGTGTTTCTGATGAGCTGCCCTCCGG

M7-6
GGTGGTGCTACTGACGGCGACGGCGAGGGAGACGGTTCGCGGGAAGCTG
GAGATGGCGCTGGCGGCGATCGAGCTGGTGGGAGCGGGGTTGGTGGT

M7-7
GCAGAGCGATCTCGATTGGCTCGTTTTTTCNGCGTCACGCAACATTTCTGCT
TTCCCCTATCATCTCGTTGACAATGGGACGCAATGGGCGGATGAACATGGC
AAACAAGTCAGCTTCAAGAAGGTATCTTTACGTGTAACCATGGCGATCAATC
ACAGATTCAGATAGATTCATAAATTCTCATTTAATCTAGTTGCTGAGCAGATG
TTCTTCCATTTCATCCATTAGTTGCAGGAGAATCAGTCTGACAAGTGGGCAG
GACATGGCAGTGACAGGGGGCTCATTGTGGCATGGAACAATCCAAGCACTC
GTATGGTTGCAGGAGTAATGCCTTCTTTTCTGAATGGAAGAGGAGTACACAA
TTGGTGGCTGATTCATGAGGTTCTCTCATCTGAAACTAGACTTGTGTTTGAT
GCAAGCAATCTTGTTCTTGGATTATATCCTGAAAATTTCAGCGAAAAGCGCA
GCACGAGCACTAGCAGAAATGTTAGTAACCCTGATGGATCCTGGGAGTATG
ATGTCAATCGACATCTAGCTGCACTTTATGGATCGTATTGCTATGAATTGCC
AAGAAGAAACTCCCCCATGGTGTATAAAGTGGTGAAGCAGTTTGAAGATTAT
ATGTTTAGTAAAAATGAAGGGCCAAACTTGTCAAATTCTGTCATAAACAAAGA
ACAAAATGTACATCCAGAGGGAGGTAGCCTGTGTGAAAAGGAGATCAGCTA
CTCATCAGCAGTTAATTTGCCACATTCTTTAGAAATGCTCCTTGAACTTGTTG
CTGACAAGAACAGGTCCGTTGTTCTTGCTGTGGCTGGTGCAAGTTACAGGG
ACATGCTTATGAGCTGGGTGTGCCGCTTGCGCCGTCTCCGG

M7-8
CGGGGATTGGGAACCCGATATATAGCATTTTCTTTAGGCTTTATTCGATTAAT
CACTGTGAGGGAGGGATTGGAGGGGATTTATTTCACACCTATTGTGGNGNG
GAATTATTCTCCCTCAATCTCCTCCAATCCTTTTCAATCTCTTTCAAACCGAA
CAAGCCCTTACACAATTAGTTTCGAGCTCTGAAGAGTTATCCTAGGCTGGAA
ATGGCGCCATAATCTCCAATTAATTGGCTCTCTTAACCGTACGTACCCAGTA
CCCTGGCCATTAGTACCATTGATGTAATTCGCAGTTNNAAANNGTAGCCTCT
GCCTCTCTAGCAGGCAGCTGGTGACCCTGAGCTAGC

M7-9
CCGCCTGCGTCTGCATCCACAGAGACGANAATTCCCAAGTTTCAGAGATGA
TGAACTAGTGCTCTGAACAATGGCAAAAGTTCAGAGTTTCAGAGATGATACG
ATGCTTTGCAGGTGAAAATGCAGATTATTACCTGGTTTGCACAGGGTGTTGG
TTGGTCACAGTAGTACTGATCGATGATGATTGGATTGGAGACGTTCTTCATC
ACGATGCCCTCGAACCTCAGGTTGTGAACATACCCCATCCCTCCCTGATCN
AGCACAAAGTTGCATTTTGTCATATAATTACTCCTTTTTCTGAATAGATTGTTC
TTGTCAGAACCAAGAACTGCAAGTGTTTTTTCCCCTCATCAGATTGTTCTTGT
GAGAATTCAGAAGTGTGATGTCCTGCAACTAACCTGCCAACTCTTGATCCGT
ACACCGTTGGTTGTGTTTGTCAGCAAGCAGGTGTCCACCCTCACGTTCTCTA
TTCTGTCAGTGGTCCGG

M7-10
ATATGGCAATACAGCTGANGTTCCTTTTGCTGTANAANAATTTCAGTATTTCT
TTTCCTGAAAAACGTGTTCACCAATATTGAGTCACCTAGAAAGGTTGAGCGG
TCTTCAGGCTAGGCGGCTAGCACCATAAGGTGGTGGGCTTGCCAACCTAG
GTTCGAGCCTCACTTTTCTTAATAAATTCCGATATGAGGGCTCCTCCTCTCG
TATCCAGTGTTTTGTTTAATATTCAGTCACTCAAGACAACCTACAGCCAATTT
GAAACAAGTTCATACAAATGAAATTTGTGTACCTCCTGACTCTTTGGCCTCTT
TTTTGGCGCCATTTGGCTCACTTTGAGCAGGACCAAGGGCTGTTTTCAGAAA
AAGATGGGTTGCCAAGGAAAGCGTTTCCAAACGGTGGAAGGGCGAGAGG
GGTCTATACTCCGGGGCATTCTCCCGTCGTGGCCTGATGGGAACGGCTGC
CGATGCAAAAAAGATTGCTCATGACATTCCTATGCAATGGAAGTCGTCCAAA
GGGAGGAGTCGTCCAGCCAAACCTTCTCCTTAGCTCTGTTGTANATGCTCC
ATGGATGGCGAGTGTGTGTACCGTCCTGCCGTTTTTGGACCTGTTTTGTTTT
TGATTG

M7-11 (on construct)
AGAGAGACTGGTGATTTCAGCGTGTCCTCTCCNAATGNNNTGAACTTCCTTA
TATAGAGGAAGGGTCTTGCGAAGGATAGTGGGATTGTGCGTCATCCCTTAC
GTCAGTGGAGATATCACATCAATCCACTTGCTTTGAAGACGTGGTTGGAAC
GTCTTCTTTTTCCACGATGCTCCTCGTGGGTGGGGGTCCATCTTTGGGACC
ACTGTCGGCAG

M7-12 (on construct)
GCGCGGGGAGAGGCGGTTTGCGTATTGGCTAGAGCAGCTTGCCAACATGG
TGGAGCACGACACTCTCGTCTACTCCAAGAATATCAAAGATACAGTCTCAGA
AGACCAAAGGGCTATTGAGACTTTTCAACAAAGGGTAATATCGGGAAACCTC
CTCGGATTCCATTGCCCAGCTATCTGTCACTTCATCAAAAGGACAGTAGAAA
AGGAAGGTGGCACCTACAAATGCCATCATTGCGATAAAGGAAAGGCTATCG
TTCAAGATGCCTCTGCCGACAGTGGTCCCAAAGATGGACCCCCACCCACGA
GGAGCATCGTGGAAAAAGAAGACGTTCCAACCACGTCTTCAAAGCAAGTGG
ATTGATGTGAACATGGTGGAGCACGACACTCTCGTCTACTCCAAGAATATCA
AAGATACAGTCTCAGAAGACCAAAGGGCTATTGAGACTTTTCAACAAAGGGT
AATATCGGGAAACCTCCTCGGATTCCATTGCCCAGCTATCTGTCACTTCATC
AAAAGGACAGTAGAAAAGGAAGGTGGCACCTACAAATGCCATCATTGCGAT
AAAGGAAAGGCTATCGTTCAAGATGCCTCTGCCGACAGTGGTCCCAAAGAT
GGACCCCCACCCACGAGGAGCATCGTGGAAAAAGAAGACGTTCCAACCAC
GTCTTCAAAGCAAGTGGATTGATGTGATATCTCCACTGACGTAAGGGATGAC
GCACAATCCCACTATCCTTCGCAAGACCCTTCCTCTATATAAGGAAGTTCAT
TTCATTTGGAGAGGACACGCTGAAATCACCAGTCTCTCTCTACAAATCTATC
TCTCTCGAGCTTTCGCAGATCCGGGGGCAATGAGATATGAAAAAGCCTGA
ACTCACCGCGACGTCTGTCGAGAAGTTTCTGATCGAAAAGTTCGACAGCGT
CTCCGACCTGATGCAGCTCTCGGAGGGCGAAGAATCTCGTGCTTTCAGCTT
CGATGTAGGAGGGCGTGGATATGTCCTGCGGGTAAATAGCTGCGC

M7-13 (on construct)

CTTGGCTAACATAAGAAGCCATATAAGTCTACTAGCACACATGACACAATAT
AAAGTTTAAAACACATATTCATAATCACTTGCTCACATCTGGATCACTTAGCA
TGCATAAACTATTACAACCAAGGCTCATCTGTCAACAAACATAAGACACATT
GCTCATGGAGAGGAGCCACTTGCTACATCTTCATTATTCTTAGAAAATTCTAT
TGCGTCTTCATCCTGTTAATACACAAAAATAAGTCAGTTTTGGATAAATAAAT
ACATATAGAAGAACATGAATTGATATGCAGGGAGTATAAATAAATACATATAG
GAGAACATGAATCTGTGAACTAACACGGCTGGGAGCTAGGCAGCTAGCAGC
TAGCGC

M7-14 (fused ends on construct)
AATCGGTTATACGATAACGGTCGGTACGGGATTTTCCCATCCTACTTTCATC
CCTGGATGGATGAAAACGGTCGGTAACGGTCGGTAAAATACCTCTACCGTT
TTCATTTTCATATTTAACTTGCGGGACGGAAACGAAAACGGGATATACCGGT
GAAACGGTCGGG

M7-15 (on construct)
GCGCAGCTATTTACCCGCAGGACATATCCACGCCCTCCTACATCGAAGCTG
AAAGCACGAGATTCTTCGCCCTCCGAGAGCTGCATCAGGTCGGAGACGCT
GTCGAACTTTTCGATCAGAAACTTCTCGACAGACGTCGCGGTGAGTTCAGG
CTTTTTCATATCTCATTGCCCCCCGGATCTGCGAAAGCTCGAGAGAGATAGA
TTTGTAGAGAGAGACTGGTGATTTCAGCGTGTCCTCTCCAAATGAAATGAAC
TTCCTTATATAGAGGAAGGGTCTTGCGAAGGATAGTGGGATTGTGCGTCAT
CCCTTACGTCAGTGGAGATATCACATCAATCCACTTGCTTTGAAGACGTGGT
TGGAACGTCTTCTTTTTCCACGATGCTCCTCGTGGGTGGGGGTCCATCTTTG
GGACCACTGTCGGCAGCGACGGCAGAGGCATCTTGAACGATAGCCTTTCCT
TTATCGCAATGATGGCATTTGTAGGTGCCACCTTCCTTTTCTACTGTCCTTTT
GATGAAGTGACAGATAGCTGGGCAATGGAATCCGAGGAGGTTTCCCGATAT
TACCCTTTGTTGAAAAGTCTCAATAGCCCTTTGGTCTTCTGAGACTGTATCTT
TGATATTCTTGGAGTAGACGAGAGTGTCGTGCTCCACCATGTTCACATCAAT
CCACTTGCTTTGAAGACGTGGTTGGAACGTCTTCTTTTTCCACGATGCTCCT
CGTGGGTGGGGGTCCATCTTTGGGAC

M9-1
TTATCTGAAGATCTAACAAGATGAAAGAGGAACTGATTCTGCGTTTCCGAAC
AGTGAACGGTGCCTGCATTTGTGTTTCTTCTTTGCAAACCGAAGCTAGTACA
TTATACTCTGCTACTACTTTAAGCACTGAGACAATGTCATAGTTAAATGTTCA
ATGCTCCTTTCAGTATATTAGTATTAAGTGCTAAAAATAACTGAAAAGACATA
ACTATAGAATGCAGCCGCAAAGCCTGCATCAAAGGACCTAAACGCCAGCTT
AAATAGAAAGACAATTTGGTAAGTTCGATTCCAACGTGATACATTAACTCCTT
GACCCAATTCCATGTGATGCCAAACCGACCGAACGGCTTACGCGATTTAAC
AAAGATTCATCTTTGCGATTGAAAAATGCATGTGAATAAAAAGCAGAGGCCT
CACATGCTCAGCTTAACGGTTATCTTTCAGTTCAGAGCCAAACCCTGCATGG
CTGCGTATAGCTGAAAAATGAGACGGNAATAAAATTGCAGCTGAATCCAGAT
ACCATTTGGTATTTTAGCAATTGACATTAACTCGCATCCAAATCAAGGTCTAA
TTAACATAAGTTTATATGGTTGCTGCTTGTCGCTTAAGAATCAGGAAAGCAA

CCTCTGAATTTGGAGGTAATCCATGAAAGTGAGGTTGTGTAGACATTTGATT
TGAAAGCTAATAACTAAATTTTACTGAATATTTCACCTAAACACAATTCCATTC
CATTGTGAAACGATCCCTATACATCATGTACGCAACCTACCAAAAACTAAGA
AGAATGATGTCAAACAAATCCGCATTGTCCTGATCACTAATCTTCCAGGAAT
TTCCCTCCAGAGAAGATCAAAAGCACGGCGTCAGTACGTTGAGCAGTGACA
CCCTCGCGTTCACGAGCGCCCGG

M9-2 (fused ends on construct)
TAACAAAATCGGTTATACGATAACGGTCGGTACGGGATTTTNNCANNCTACT
TTCATCCCTGAGAGCTAGGGATGAAAACGGTCGGTAACGGTCGGTAAAATA
CCTCTACCGTTTTCATTTTCATATTTAACTTGCGGGACGGAAACGAAAACGG
GATATACCGG

M9-3
CAAGGAAGGTGAGGAGGAAGCCGAGCAAGCTGCAGCCGGCGAGCGC
